# Supplementary material for: The pentaglycine bridges of Staphylococcus aureus peptidoglycan are essential for cell integrity
Source: Sci Rep. 2019 Mar 21;9:5010. doi: 10.1038/s41598-019-41461-1 (PMC6428869; doi:10.1038/s41598-019-41461-1)
Supplement: Supplementary file 1 — Supplementary information [file 41598_2019_41461_MOESM1_ESM.docx]

**The pentaglycine bridges of *Staphylococcus aureus* peptidoglycan are essential for cell integrity**

João M. Monteiro^1^, Gonçalo Covas^1,3^, Daniela Rausch^2^, Sérgio R. Filipe^3,1^, Tanja Schneider^2^, Hans-Georg Sahl^2^ and Mariana G. Pinho^1,*^

1- Instituto de Tecnologia Química e Biológica António Xavier, Universidade Nova de Lisboa, Oeiras, Portugal

2-Institute of Pharmaceutical Microbiology, University of Bonn, 53115 Bonn, Germany

3-UCIBIO-REQUIMTE, Departamento de Ciências da Vida, Faculdade de Ciências e Tecnologia, Universidade Nova de Lisboa, Caparica, Portugal

*Correspondence and requests for materials should be addressed to M.G.P. (email: [mgpinho@itqb.unl.pt](mailto:mgpinho@itqb.unl.pt))


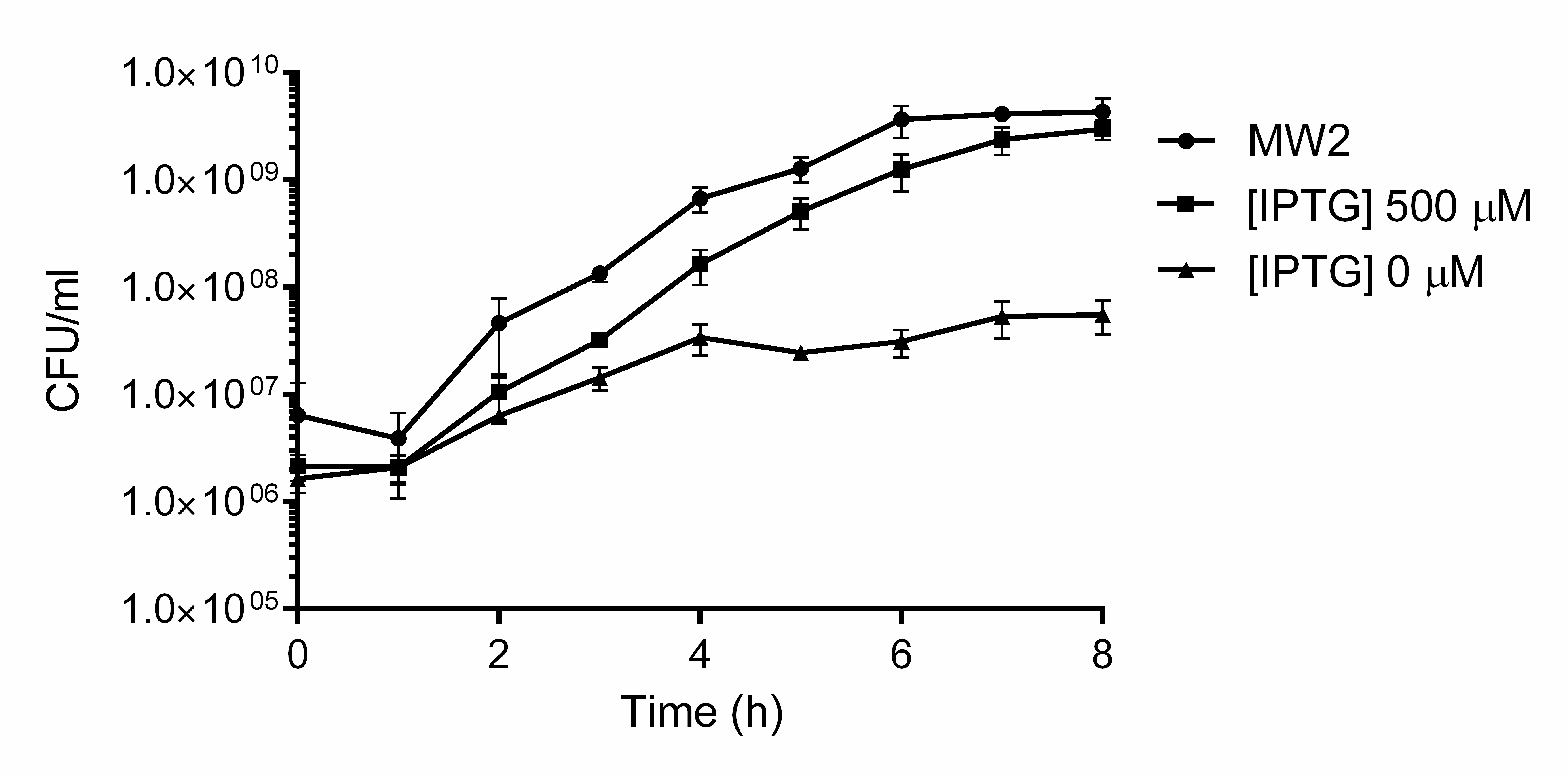


**Supplementary Figure 1**. **FemAB is essential for the viability of MW2.** Colony forming units (CFU) were counted in cultures of parental strain MW2 and of strain MW2-iFemAB grown in the presence ([IPTG] 500 µM) or absence ([IPTG] 0 µM) of *femAB* expression. Data shown are representative of three independent experiments. Symbols indicate means and error bars indicate standard deviation from six technical replicates.


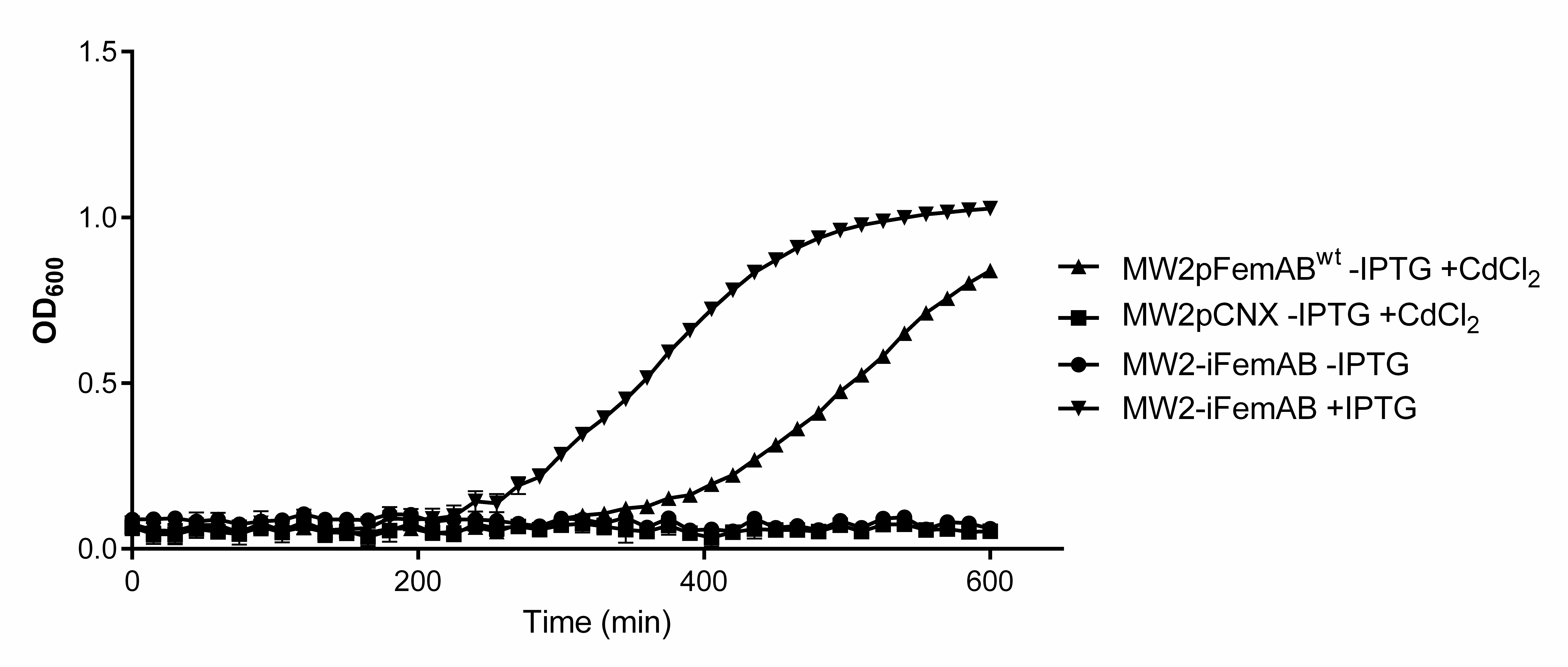


**Supplementary Fig. 2. Endogenous FemAB can be depleted in cells producing plasmid-encoded FemAB*.*** Growth curves of strain MW2-iFemAB grown in the presence (MW2-iFemAB + IPTG) or absence (MW2-iFemAB – IPTG) of endogenous *femAB* expression; and of MW2-iFemAB depleted of endogenous *femAB* expression and either carrying wild-type *femAB* in a multicopy plasmid under the control of P*cad* (MW2pFemAB^wt^ –IPTG +CdCl_2_), or empty vector (MW2pCNX –IPTG +CdCl_2_). Symbols indicate means and error bars indicate standard deviation from three biological replicates.


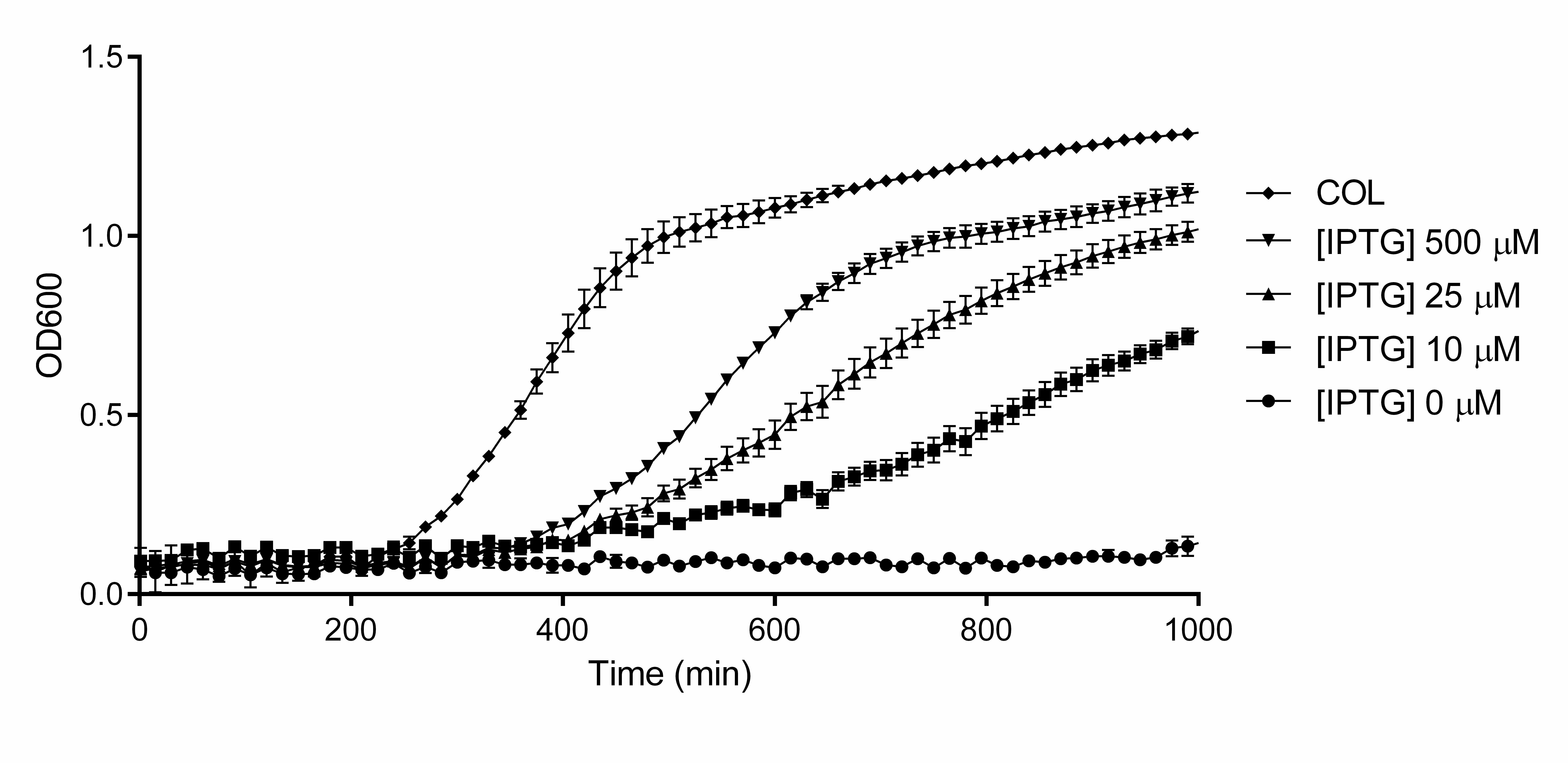
**Supplementary Figure 3. *femAB* is essential in COL.** Growth curves of COL-iFemAB with IPTG-inducible *femAB* operon. In the absence of IPTG ([IPTG] 0 µM), no growth was detected. Growth was rescued with increasing concentrations of IPTG. Symbols indicate means and error bars indicate standard deviation from three biological replicates.


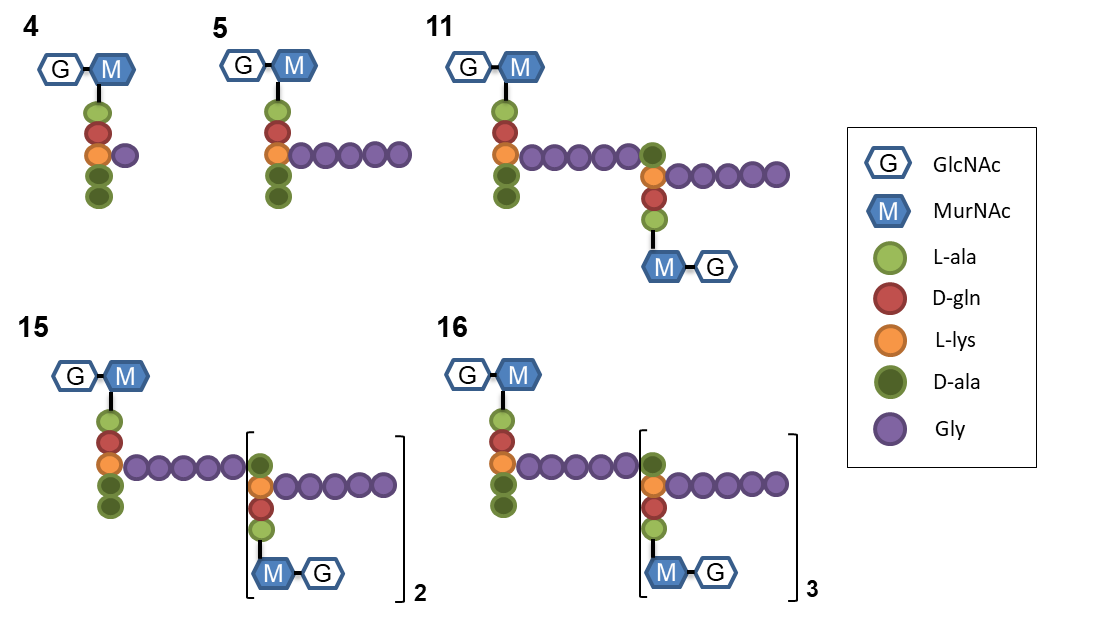


**Supplementary Figure 4. Chemical structures of muropeptide species.** Proposed structures of the peaks present in muropeptide chromatograms, according to de Jonge and Tomasz^1^. Muropeptides are numbered according to increasing retention times. GlcNAc – *N*-acetylglucosamine, MurNAc – *N*-acetylmuramic acid.


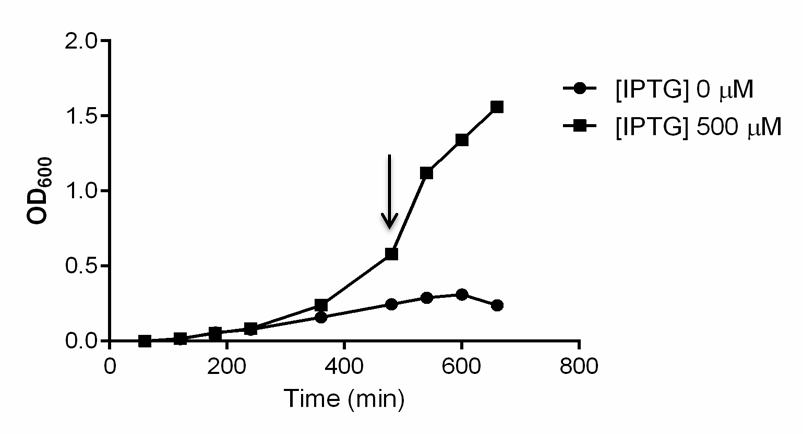


**Supplementary Figure 5. Depletion of FemAB led to growth arrest**. Growth curves of MW2-iFemAB in the presence ([IPTG] 500 µM) or absence ([IPTG] 0 µM) of IPTG, to determine the timing of growth arrest in the non-induced condition (see Methods). Cells were collected for microscopy at the indicated time point (black arrow).


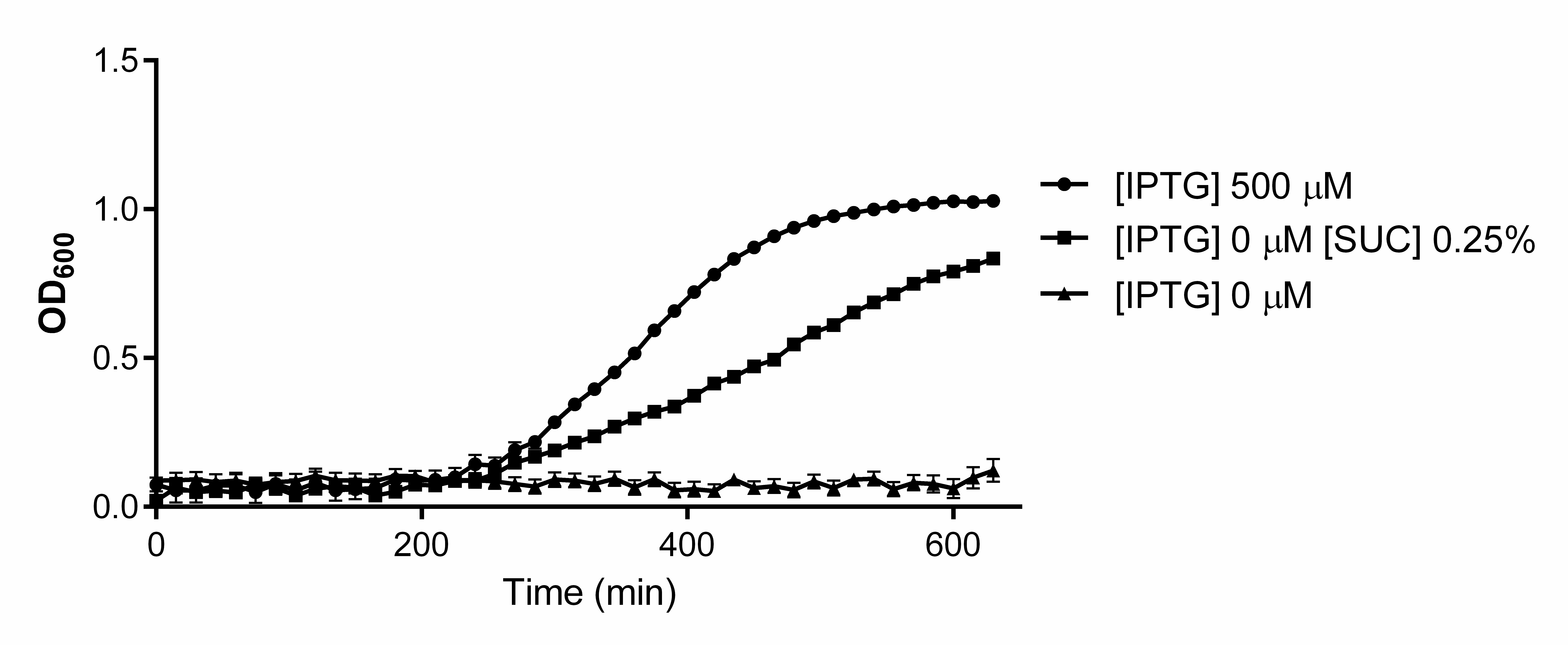


**Supplementary Fig. 6**. **Sucrose can compensate for a FemAB deficit**. Growth rates of MW2-iFemAB incubated in the presence ([IPTG] 500 µM) or absence ([IPTG] 0 µM) of IPTG, or in the absence of IPTG with 0.25% of sucrose ([IPTG] 0 µM [SUC] 0.25%). Adding sucrose to the medium allowed for cell growth in the absence of FemAB expression. Symbols indicate means and error bars indicate standard deviation from three biological replicates.


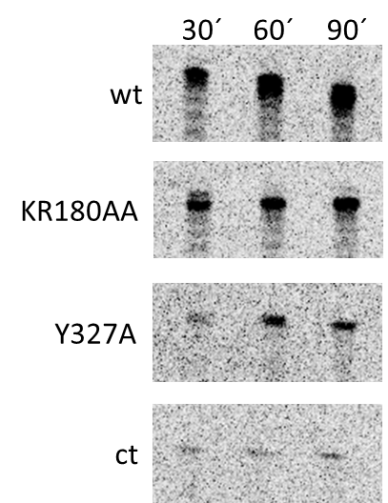


**Supplementary Fig. 7**. **Selected mutations reduce FemA activity *in vitro*.** Recombinant FemA^wt^, FemA^KR180AA^ and FemA^Y327A^ were incubated with lipid II-Gly_1_ in the presence of [U-^14^C]-glycine charged tRNA, for either 30, 60 or 90 minutes and reaction products were separated by TLC. No FemA was added to control row (ct). Figure shows representative bands that were excised for radioactivity measurements (see Methods).

**Supplementary Table 1 – Strains and plasmids used in this study.**

| **Strains** | **Description** | **Source or reference** |
| --- | --- | --- |
| *E. coli* |  |  |
| DC10B | Δ*dcm* in the DH10B background; Dam methylation only | 2 |
| BL21 (DE3)  BL21-FemX | B F^−^ *dcm ompT hsdS*(r_B_^−^ m_B_^−^*) gal* λ(DE3)  BL21(DE3) expressing full length C-ter His-tagged FemX; Kan^r^ | Stratagene  This work |
| BL21-GlyS  BL21-FemA^wt^ | BL21(DE3) expressing full length C-ter His-tagged GlyS; Kan^r^  BL21(DE3) expressing full length C-ter His-tagged FemA; Kan^r^ | This work  This work |
| BL21-FemA^KR180AA^ | BL21(DE3) expressing mutant C-ter His-tagged FemA^KR180AA^; Kan^r^ | This work |
| BL21-FemA^RF220AA^ | BL21(DE3) expressing mutant C-ter His-tagged FemA^RF220AA^; Kan^r^ | This work |
| BL21-FemA^Y327A^ | BL21(DE3) expressing mutant C-ter His-tagged FemA^Y327A^; Kan^r^ | This work |
| *S. aureus* |  |  |
| RN4220 | Restriction-deficient derivative of NCTC8325-4 | 3 |
| MW2 | CA-MRSA; SCC*mec* type IV, ST1, pbla^+^ | 4 |
| MW2-iFemAB | MW2 *femAB*::pFemABi pMGPII; Ery^r^ Cm^r^ | This work |
| MW2pCNX | MW2 *femAB*::pFemABi pMGPII pCNX; Ery^r^ Cm^r^ Kan^r^ | This work |
| MW2pFemAB^wt^ | MW2 *femAB*::pFemABi pMGPII pFemAB^wt^; Ery^r^ Cm^r^ Kan^r^ | This work |
| MW2pFemA^KR180AA^ | MW2 *femAB*::pFemABi pMGPII pFemA^KR180AA^B; Ery^r^ Cm^r^ Kan^r^ | This work |
| MW2pFemA^RF220AA^ | MW2 *femAB*::pFemABi pMGPII pFemA^RF220AA^B; Ery^r^ Cm^r^ Kan^r^ | This work |
| MW2pFemA^Y327A^ | MW2 *femAB*::pFemABi pMGPII pFemA^Y327A^B; Ery^r^ Cm^r^ Kan^r^ | This work |
| COL | HA-MRSA | 5 |
| COL-iFemAB | COL *femAB*::pFemABi pMGPII; Ery^r^ Cm^r^ | This work |

| Plasmids | Description | Source or reference |
| --- | --- | --- |
| pMUTIN4 | *S. aureus* integrative vector containing an IPTG-inducible P*spac* promoter; Amp^r^ Ery^r^ | 6 |
| pMGPII | *S. aureus* replicative plasmid containing *lacI*; Amp^r^ Cm^r^ | 7 |
| pCNX | Replicative vector containing a cadmium inducible P*cad* promoter; Amp^r^ Kan^r^ | 8 |
| pFemABi | pMUTIN4 derivative containing a *femA* DNA fragment under the control of P*spac;* Amp^r^ Ery^r^ | This work |
| pMADfemAmch | Vector containing a *femA-mCherry-STOP-femB* DNA fragment; Amp^r^ Ery^r^ | 9 |
| pFemAB^wt^ | pCNX derivative encoding a FemA-mCherry fusion and FemB, both under the control of P*cad;* Amp^r^ Kan^r^ | This work |
| pFemA^KR180AA^B | pCNX derivative encoding a mutant FemA(K180A, R181A)-mCherry fusion and FemB, both under the control of P*cad;* Amp^r^ Kan^r^ | This work |
| pFemA^RF220AA^B | pCNX derivative encoding a mutant FemA(R220A, F224A)-mCherry fusion and FemB, both under the control of P*cad;* Amp^r^ Kan^r^ | This work |
| pFemA^Y327A^B | pCNX derivative encoding a mutant FemA(Y327A)-mCherry fusion and FemB, both under the control of P*cad;* Amp^r^ Kan^r^ | This work |
| pET-24b | *E. coli* replicative vector for the expression of proteins with a His_6_ fusion at the C-terminus, under P*spac* promoter; Kan^r^ | Novagen |
| pET-GlyS | pET-24b derivative encoding a GlyS-His_6_ fusion; Kan^r^ | 10 |
| pET-FemX | pET-24b derivative encoding a FemX-His_6_ fusion; Kan^r^ | 10 |
| pET-FemA^wt^ | pET-24b derivative encoding a FemA-His_6_ fusion; Kan^r^ | This work |
| pET-FemA^KR180AA^ | pET-24b derivative encoding a mutant FemA(K180A, R181A)-His_6_ fusion; Kan^r^ | This work |
| pET-FemA^RF220AA^ | pET-24b derivative encoding a mutant FemA(R220A, F224A)-His_6_ fusion; Kan^r^ | This work |
| pET-FemA^Y327A^ | pET-24b derivative encoding a mutant FemA(Y327A)-His_6_ fusion; Kan^r^ | This work |

Amp, ampicillin; Kan, kanamycin; Ery, erythromycin; Cm, chloramphenicol

**Supplementary Table 2. Oligonucleotides used in this study.**

| **Primer Name** | **Sequence (5’-3’)** | |
| --- | --- | --- |
| spacfemab_P1 EcoRI | | GCGCGAATTCATGAAGTTTACAAATTTAACAGC |
| spacfemab_P2 BamHI | | CGCGGGATCCTTATCAAAGAACCAATCATTACCAGCATTAC |
| pcnfemab_P1 BamHI | | GCGCGCGGATCCGCAAATACGGAAATGAAATTAATTAACG |
| pcnfemab_P2 EcoRI | | CGCGCGGAATTCCTATTTCTTTAATTTTTTACGTAATTTATC |
| fema_kr180aa_fw | | ATGGACTTAGAGCAGCAAACACGAAAAAAGTTAAAAAGAATG |
| fema_kr180aa_rev | | TTTTCGTGTTTGCTGCTCTAAGTCCATCCATATTTTTAATGATG |
| fema_rf220aa_fw | | GCTGATGCTGATGACAAAGCTTACTACAATCGCTTAAAATATTAC |
| fema_rf220aa_rev | | GTAGTAAGCTTTGTCATCAGCATCAGCAAAAGCTTTTGATTC |
| fema_Y327a_fw | | GAAGTTGTTGCTTATGCTGGTGGTACATCAAATGCATTCC |
| fema_Y327a_rev | | ACCAGCATAAGCAACAACTTCAAATGGATTGATAAAGAAG |
| femaexpress_P1 BamHI | | CGCGCGGATCCATGAAGTTTACAAATTTAACAGCTAAAGAGTTTG |
| femaexpress_P2 EcoRI | | CGCGCGAATTCCTAAAAAATTCTGTCTTTAACTTTTTTAAGTGC |

Underlined sequences correspond to restriction sites

**References**

1 de Jonge, B. L., Chang, Y. S., Gage, D. & Tomasz, A. Peptidoglycan composition of a highly methicillin-resistant *Staphylococcus aureus* strain. The role of penicillin binding protein 2A. *J Biol Chem* **267**, 11248-11254 (1992).

2 Monk, I. R., Shah, I. M., Xu, M., Tan, M. W. & Foster, T. J. Transforming the untransformable: application of direct transformation to manipulate genetically *Staphylococcus aureus* and *Staphylococcus epidermidis*. *mBio* **3**, e00277-11 (2012).

3 Nair, D. *et al.* Whole-genome sequencing of *Staphylococcus aureus* strain RN4220, a key laboratory strain used in virulence research, identifies mutations that affect not only virulence factors but also the fitness of the strain. *J Bacteriol* **193**, 2332-2335 (2011).

4 Baba, T. *et al.* Genome and virulence determinants of high virulence community-acquired MRSA. *Lancet* **359**, 1819-1827 (2002).

5 Gill, S. R. *et al.* Insights on evolution of virulence and resistance from the complete genome analysis of an early methicillin-resistant *Staphylococcus aureus* strain and a biofilm-producing methicillin-resistant *Staphylococcus epidermidis* strain. *J. Bacteriol.* **187**, 2426-2438 (2005).

6 Vagner, V., Dervyn, E. & Ehrlich, S. D. A vector for systematic gene inactivation in *Bacillus subtilis*. *Microbiology* **144**, 3097-3104 (1998).

7 Pinho, M. G., Filipe, S. R., de Lencastre, H. & Tomasz, A. Complementation of the essential peptidoglycan transpeptidase function of penicillin-binding protein 2 (PBP2) by the drug resistance protein PBP2A in *Staphylococcus aureus*. *J Bacteriol* **183**, 6525-6531 (2001).

8 Monteiro, J. M. *et al.* Cell shape dynamics during the staphylococcal cell cycle. *Nat Commun* **6**, 8055 (2015).

9 Monteiro, J. M. *et al.* Peptidoglycan synthesis drives an FtsZ-treadmilling-independent step of cytokinesis. *Nature*, **554** 528-532 (2018).

10 Schneider, T. *et al.* *In vitro* assembly of a complete, pentaglycine interpeptide bridge containing cell wall precursor (lipid II-Gly5) of *Staphylococcus aureus*. *Mol Microbiol* **53**, 675-685 (2004).
